# Supplementary material for: Oral intake of Lactiplantibacillus pentosus LPG1 Produces a Beneficial Regulation of Gut Microbiota in Healthy Persons: A Randomised, Placebo-Controlled, Single-Blind Trial
Source: Nutrients. 2023 Apr 17;15(8):1931. doi: 10.3390/nu15081931 (PMC10144437; doi:10.3390/nu15081931)
Supplement: Supplementary file 1 [file nutrients-15-01931-s001.zip › Table S1_Inclusion&Exclusion criterias.pdf]

Table S1. Randomised, placebo-controlled, single-blind trial to study the effect of oral intake of Lactobacillus plantarum LPG1 on the human gut microbiota. Inclusion and exclusion criteria of the participants (volunteers) in the clinical trial.

Inclusion criteria

1. Informed consent: All participants agreed to be included in the study by signing the protocol approved by the Reina Sofia University Hospital Clinical Research Ethics Committee. In this written statement of consent, it was stated that patients were chosen for inclusion in the groups on a random basis.
2. Diagnostic criteria. Recruitment was carried out among the general population without any disabling diseases or whose severity implies a lifespan of fewer than five years, who present:
   - Gender: men and women
   - Age: >20 but <76 years old
   - Body mass index (weight/height^2): 20-28
   - Medical history: absence of current disease
   - Normal physical examination
   - Normal electrocardiogram
   - Laboratory tests (haematology, biochemistry, coagulation, urine and stool) without alterations.

Exclusion criteria

1. Patients with established cardiovascular disease or high coronary risk.
2. Patients with limitations to follow the protocol: People with no personal or family capacity to adhere to the indicated dietary protocol for any reason will be excluded.
3. Severe or difficult-to-control risk factors: subjects with hypertension and diabetes with organ involvement that limits their survival (chronic renal failure with creatinine persistently >2 mg/dl) and incapacitating clinical manifestations of cerebral arteriosclerosis will be excluded.
4. Chronic diseases not related to coronary risk: severe psychiatric conditions, chronic processes requiring treatment such as chronic renal failure, chronic liver disease, neoplasms under treatment, chronic obstructive pulmonary disease, endocrinopathies susceptible to decompensation and diseases of the gastrointestinal tract.
5. Participants in other clinical trials at the time of selection or in the 30 days prior to the start of the trial.
6. Pharmacological treatment (particularly antibiotics) of any type 4 weeks prior to the study.
7. Use of any pre- or probiotic as a dietary supplement in the 3 months prior to the study.
8. Consumption of any type of olives or fermented vegetables (capers, fermented cabbage, pickles, etc.) in the 15 days prior to the start of the study. Pharmacological treatment with an unstable dose within the 4 weeks before to screening (including psychotropic and other drugs affecting the alertness and cognitive capacity of the patients).
9. Alcohol and drug abuse.
10. Pregnant or lactating women.
